# Malaria risk in Corsica, former hot spot of malaria in France

**DOI:** 10.1186/1475-2875-9-231

**Published:** 2010-08-12

**Authors:** Céline Toty, Hélène Barré, Gilbert Le Goff, Isabelle Larget-Thiéry, Nil Rahola, Daniel Couret, Didier Fontenille

**Affiliations:** 1 Institut de Recherche pour le Développement, UR016, 911 avenue Agropolis, BP64501, 34394, Montpellier, cedex 5, France; 2 CNRS UMR 6134, Université de Corse, Faculté des Sciences et Techniques, Laboratoire Parasites et Ecosystèmes Méditerranéens, BP52, 20250 Corte, France; 3 Direction de la Solidarité et de la Santé de Corse et de Corse-du-Sud/Immeuble Castellani/Quartier St-Joseph/BP 413/20305 Ajaccio Cedex 1, France; 4 Institut Pasteur, Plate Forme CEPIA, Département de Parasitologie et Mycologie, 25 rue du Dr Roux, 75724 Paris cedex 15, France

## Abstract

**Background:**

The prevalence of *Plasmodium falciparum *and *Plasmodium vivax *malaria was very high in Corsica just before the Second World War. The last outbreak was in 1972 and the most recent indigenous case was in 2006.

**Results:**

Analysis of historical data shows that anopheline vectors were abundant. Recent surveys demonstrated that potential vectors are still present in Corsica, despite the likely disappearance of *Anopheles sacharovi*. Moreover, *P. falciparum *can develop experimentally into these mosquitoes, notably *Anopheles labranchiae*, which is locally abundant, and parasites are regularly introduced into the island.

**Discussion, Conclusions:**

The presence of vectors, the introduction of parasites and the conducive climate raise questions about the possibility of malaria re-emerging and becoming re-established in Corsica. Analysis of historic and current parasitological and entomological data shows that the current theoretical risk of indigenous cases or malaria foci is negligible, particularly since there is very little contact between humans and *Anopheles *mosquitoes, *Plasmodium *carriers are reliably treated and there is a widespread vector control on the island.

## Background

Climate and environmental changes coupled with increasing intercontinental traffic raise the spectre of the emergence or re-emergence of a number of diseases, which had been eliminated or are under control in temperate Europe [1,2]. In Italy in 2007, there were more than 200 cases of Chikungunya fever [3], which is caused by a virus transmitted by *Aedes albopictus *and *Aedes aegypti*. Foci of West Nile virus infection (transmitted by *Culex *mosquitoes) are regularly reported around the Mediterranean rim [4]. Cases of imported and airport malaria, as well as occasional indigenous cases, have been documented in Europe [5]. By virtue of its southern latitude, its recent history of malaria cases and the large numbers of tourists visiting the island, Corsica, a Mediterranean region of France, warrants evaluation with regards to the risk of introduction, emergence and establishment of such diseases, in particular malaria. On the basis of historic information and recent data acquired between 2002 and 2009, this article reviews malaria and potential *Plasmodium *vectors in Corsica initiating a malaria risk assessment.

### Corsica and its history of malaria

**Figure 1:**
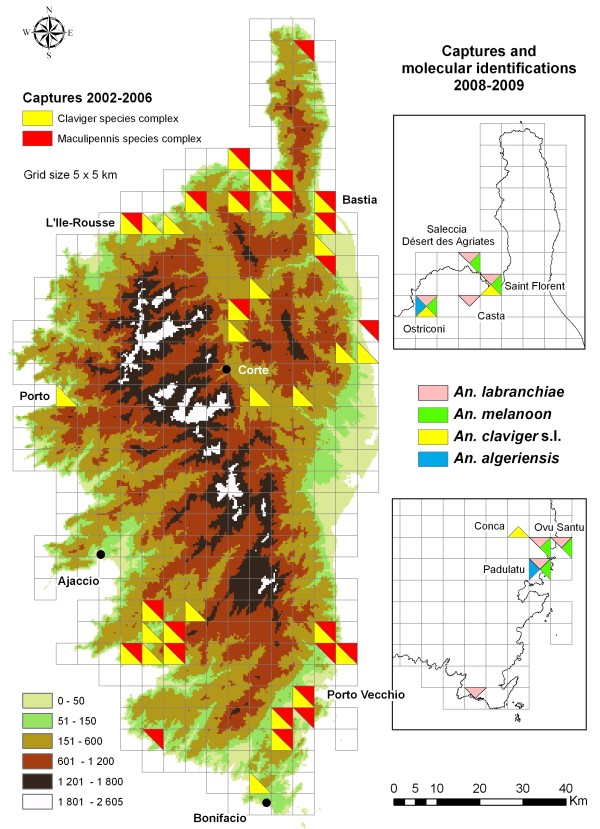
**distribution of anophelines belonging to the Claviger and Maculipennis species complexes, based on data captured between 2002 and 2009**.

Corsica, to the south of mainland France, is the most mountainous island in the Mediterranean. Its high elevation creates a damp environment and induces heavy precipitation, resulting in many rivers. Its geographical location and relief give rise to markedly contrasting temperatures and rainfall (Figure 1). In Corsica, precipitation through the year is very irregular with two peaks, one in November and December and the other in February and March. Less than 30% of the rain falls between April and September, a period which coincides with the highest temperatures. The coast is characterized by clement temperatures (an annual mean of 17°C with mild winters and hot summers) and moderate precipitation (600 - 800 millimetres per annum) with very dry summers. Between altitudes of 600 and 1,200 metres, seasonal variations are more marked with harder winters and heavier precipitation. Above 1,200 metres, the winter is long and cold with greater precipitation (1,200 - 2,000 millimetres per annum).

*Plasmodium vivax *and *Plasmodium falciparum *were endemic in Corsica until after the Second World War, with high parasite prevalence in some regions: 14.7% for the island as a whole, up to an average of 26.2% on the east coast in the autumn of 1921, and 23.4% in children (n = 1845) sampled across the entire island in 1947 [6,7]. From the beginning of the 20^th ^century, a vector control programme based on environmental improvements (notably the draining of marshes) with measures directed against larvae (the introduction of fish and the application of chemicals to larval development sites) and adults (mechanical control followed by indoor insecticide spraying using several compounds such as DDT, gamma-hexachlorocyclohexane (HCH) (lindane^®^), deltamethrine, cypermethrin), coupled with systematic quinine administration to the population, led to greatly reduced prevalence and ultimate eradication of the disease [8].

With not one single case of malaria reported between 1953 and 1964, Corsica was said *'to have Anopheles without malaria' *and measures to control this mosquito were somewhat abandoned for those to control other mosquitoes--*Aedes *and *Culex*--which represented a significant problem to tourism. However, between 1965 and 1972, *P. vivax *malaria re-emerged with the arrival of immigrants from endemic regions of North Africa, with 31 cases (including 20 indigenous cases) in the north of the island in 1970. In 1971, 19 new cases were reported, including 10 indigenous cases with increased foci. Finally, two new indigenous cases were reported in 1972 [9]. The epidemic was not fully controlled until 1973 through the treatment of patients and indoor insecticide spraying operations [10]. The malaria control agencies were remobilized and implemented further larvicide treatments. Prophylactic drug administration and insecticide treatment of adult mosquitoes were abandoned in the 1980s.

In August 2006, one case of indigenous *P. vivax *malaria was diagnosed in the Porto region (in the south of the island) [11]. Although the vector control agencies located and treated an *Anopheles claviger *s.l. larval habitat in the area at the end of July 2006, the anopheline vector species was never definitively identified. This case, occurring in a region which had historically been at low risk for malaria, indicates that the island is still vulnerable to parasites and that vigilance is warranted.

### Historical and potential vectors

#### Distribution

Of the thirteen anopheline species identified in Corsica [12], only two are believed to play a significant role in indigenous malaria transmission [13], namely *Anopheles labranchiae *and *Anopheles sacharovi*. Other anopheline species which have been suspected of acting as vectors in other parts of Europe have been reported in Corsica, namely *Anopheles algeriensis, Anopheles atroparvus*, *An. claviger, Anopheles hyrcanus*, *Anopheles maculipennis *s.s., *Anopheles marteri, Anopheles melanoon*, *Anopheles messae*, *Anopheles petragnani*, *Anopheles plumbeus*, *Anopheles superpictus*, *Anopheles subalpinus *(now known to be a junior synonym of *An. melanoon *- Linton et al. 2002 [14]). However, on the basis of their physiological characteristics--in particular the zoophily of certain species--these seem to have played at most a minimal or accidental role as vectors for *Plasmodium*.

The last anopheline distribution surveys conducted in the middle of the 20^th ^century--focussing on members of the Maculipennis species complex--produced distribution maps for the whole island [7], although the identification methods used (egg morphology, larval chaetotaxy) had limitations. Four species in the Maculipennis species complex were listed following capture at twenty sites, *viz*. (in order of decreasing abundance) *An. labranchiae*, *An. sacharovi *(referred to as *Anopheles elutus)*, *An. melanoon *and *An. messae*. In the 20^th ^century, *An. labranchiae *had a strong presence around the entire coastline with expansion into the interior up deep valleys, e.g. as far as Corté (436 meters in altitude). Some experts claimed that *An. sacharovi *was abundant but not many locations were reported [13,15-19].

From 2002 to 2006, 125 larval habitats were surveyed. The collected anophelines were identified morphologically and the species identity of 0.5% of larvae of the *An. maculipennis *species complex was confirmed by multiplex polymerase chain reaction (PCR). *Anopheles labranchiae *was collected in 41 larval habitats. It is currently present in much of the open country. In particular it has colonized the focus of the last malaria outbreak recorded in 1970 - 1972 (Nebbio in the St-Florent region) and an area where malaria used to be prevalent (the region of Porto-Vecchio which is a major tourist destination in the summer). The Désert des Agriates (in the north of the island) is highly conducive and, although many tourists visit the region in summer, few people live there year-round (Figure 1). These observations were confirmed in a survey conducted in July 2008 (Table 1).

**Table 1:** Number of *Anopheles *of Maculipennis complex identified by PCR, by area and capture method, July and August 2008.

	Larvae		Mosquitoes attracted by humans		Mosquito magnet		Indoor resting mosquitoes	
	*An labranchiae*	*An melanoon*	*An labranchiae*	*An melanoon*	*An labranchiae*	*An melanoon*	*An labranchiae*	*An melanoon*
Désert des Agriates	220	10	71	1	5	0	72	6
Porto Vecchio	2	34	11	1	2	18	4	4
Ostriconi	69	38	7	1	1	0	No site found	
Saint-Florent	0	0	0	0	3	1	No site found	

*Anopheles sacharovi *has not been detected in morphological and/or molecular analyses of samples taken since 2002 and it was last identified in the 1950 s [13]. In the western Mediterranean, *An. sacharovi *was confined to certain coastal regions along the Italian peninsula, in northern Sardinia and the entire eastern edge of Corsica (In mainland Italy, the last mention of *An. sacharovi *was in 1963 in Veneto [20]. This species has never been mentioned in mainland France, Spain or even the North African coast. In Sardinia, the ERLAAS (*Ente Regionale per la Lotta Anti-Anofelica in Sardegna*) malaria control programme developed by the American army after the Second World War failed to detect a single specimen of this species despite rigorous surveillance over five successive years (1946-1950) [21]. The abundance and historical distribution of this species in Corsica, therefore, remain controversial although it was identified in entomological surveys conducted between 1947 and 1953 [13], especially close to the sea. In practice, *An. melanoon *cannot be distinguished from *An. sacharovi *on the basis of chaetotaxy of antepalmate setae of abdominal segments IV and V (setae 2-IV and 2-V) [22] as was sometimes the parameter used in Corsica. Colour, egg raft appearance and egg shell structure seem more reliable specific determinants to distinguish between different members of the Maculipennis species complex [23]. Although the possibility of residual foci of *An. sacharovi *cannot be definitively ruled out, it is likely that this species has been eradicated from the western Mediterranean as is the case in Romania [24] and in Cyprus in 1967 [24].

Analysis of the current risk of malaria will, therefore, focus on the risk associated with *An. labranchiae *which, together with the Claviger species complex, is the most common species on the island.

#### *Anopheles labranchiae *biology

Although a few one-off studies were conducted on the ecology and distribution of *An. maculipennis *s.l. from the beginning of the 20^th ^century [25-27], systematic study of the biology of *An. labranchiae *only really began with the seminal works of de Buck *et al *[28] and Martini *et al *[29] on the Maculipennis species complex, especially in Corsica [16,30] and Italy [23].

In Corsica, *An. labranchiae *larval habitats are highly diverse, from marshes to residual pools from running water and irrigation channels. The water may be fresh or brackish: *An. labranchiae *tolerates salinity of up to 3 g/l [13,22,31-33]. According to Toumanoff [7], the most productive larval habitats are permanent wetlands that do not dry out in dry weather. This species prefers well-exposed sites with relatively dense vegetation although it can also breed in shady sites with sparse vegetation [34]. *Anopheles labranchiae *is intolerant of organic and mineral pollution [24], and larvae may tolerate wide temperature variations [35]. Pre-adult stages of this species have been found in these larval habitats from June to October although Toumanoff & Rageau [17] and Jaujou [13] have reported that females can lay eggs as early as February. In Morocco, the species can lay several times a year with up to seven hatching cycles [36]. In Corsica, at least three generations in one year have been observed [37], the annual number of generations depending on local climatic conditions [38,39]. In Corsica, *An. labranchiae *hibernates in the adult form [13]. According to Sautet [39], diapause is not systematic and hibernation is induced by low temperature. Reproduction could occur during winter: in the laboratory, egg-laying has occurred during winter months at a temperature of 16°C [39]. In addition, females engorged with blood (including human blood) have been found in the middle of winter [13]. It seems impossible that eggs could survive the winter, but eggs laid at the end of the hot season have greater resistance to low temperatures and are more likely to hatch [40].

In entomological surveys conducted in 2008 in the framework of the EDEN (Emerging Diseases in a changing European eNvironment) Project, mosquitoes were sampled using various different methods (larval harvest, attracted by humans, capture of resting adults, light traps, CO2 Mosquito Magnet^® ^traps) and identified, first on the basis of morphological characteristics and then, for species belonging to the Maculipennis species complex, by species-specific PCR [41].

In various different types of larval habitat (permanent marsh, residual pools associated with streams and canals) surveyed in four locations, it was shown that, of 581 *Anopheles *identified as belonging to the Maculipennis species complex by PCR, 80.4% were *An. labranchiae *and 19.6% *An. melanoon *(Table 1), with major differences in terms of larval abundances and relative proportions at different sites, from 96% of *An. labranchiae *in the Désert des Agriates, to 6% of *An. labranchiae *among Maculipennis species complex individuals in the Porto Vecchio region.

*Anopheles labranchiae *was the only anthropophilic anopheline observed biting a human in captures conducted between June and August 2005 at Ostriconi (in the Ile-Rousse area) and in the Porto-Vecchio region. The mean bite rate per evening (i.e. two hours before to two hours after sunset) per person was low: 2.3 females at Ostriconi and 0.7 female at Porto-Vecchio.

In July 2008, human-attracted mosquito captures were performed at five sites in the south and three in the north of Corsica. *Anopheles maculipennis *s.l. (mainly *An. labranchiae *(88% to 99% of PCR identified mosquitoes, the rest being *An. melanoon*)) was biting human at four of these sites (Saleccia, Padulatu, Ostriconi and Ovu Santu) with 1.0 to 12.9 bites/person/hour. Such anthropophilic behaviour had been previously observed by Sautet [39] and, according to Bates & Hackett [22], the behaviour of *An. labranchiae *is probably predominantly opportunistic.

Immuno-assay analysis [42] of 20 blood meals in resting *An. labranchiae *females collected in a cowshed at Saleccia in July 2008 showed that they had preferentially fed on bovines (n = 19), but one individual had bitten a human. These results are consistent with those of Bailly-Choumara [43] in Morocco in May 1968, who observed that this species could feed on a variety of mammals, including humans (5.1%), but that bovines were the preferred host (82.4%, n = 1126 blood meals analysed). In contrast, in Italy in 1935, before vector control measures, Hackett & Missiroli (*in *[44]) noticed that *An. labranchiae *females tended to prefer humans but not exclusively.

In July and August 2008 and June 2009, males and females of *An. labranchiae *were found resting in almost all stone animal shelters in Désert des Agriates, sometime exceeding one hundred females per day per site, and numbers were replenished on an almost daily basis around the marsh that we was producing larvae of *An. maculipennis *s.l.

#### *Anopheles labranchiae *vectorial competence

Before this study only one experimental transmission attempt had been made with *An. labranchiae*, using Italian specimens from Tarquinia (Latium) feeding on subjects from Kisumu in Kenya carrying *P. falciparum *gametocytes [45]. Of 31 tested specimens, none was found to be carrying an oocyst between the 5^th ^and 20^th ^days of observation (n = 17), and none had sporozoites in their salivary glands from the 12^th ^day of observation.

Nevertheless, in the past this species has been an important malaria vector in the Mediterranean, especially in Italy, Sardinia and Corsica; this is because it is highly anthropophilic in the absence of indoor housing of livestock. It should be remembered that, from the early days of malaria research, scientists have documented very high sporozoite indices in anopheline populations in Corsica. Referring to anophelines in a broad sense, Léger recorded a sporozoite index of 2.37% in 1913 [25] and the Sergent brothers one of 1.20% (*in *[46]). But it is only from the mid-20^th ^century onwards that reliable species-specific infection indices have become available: naturally-infected *An. labranchiae *were found in Corsica in August 1947 on both the east (Borgo) and the west (Liamone) coasts. Then, in October 1947, two infected females *An. maculipennis *s.l. were found in a batch of 40 specimens: one in Furiani containing three young oocysts and another carrying a large number of sporozoites in Borgo. In 1949, Boyd (*in *[47]) reported a sporozoite index of 1.06% in Italy.

In October 2008 and July 2009, the competence of *An maculipennis *s.l. from Corsica for the African NF54 strain of *P. falciparum *was experimentally tested by the Pasteur Institute Anopheles Production and Infection Centre (Centre de Production et d'Infection d'Anophèles, CEPIA) in Paris. After a fast of 24-40 hours, the female mosquitoes collected in 2008 were infected with mature gametocytes. The mosquito midguts were examined on Day 8 to establish the presence of oocysts: of 99 dissections (95 *An. labranchiae*), 13 contained an oocyst and one was carrying two oocysts (all *An. labranchiae)*, i.e. a prevalence of 14% compared with 86% for control *An. gambiae*. In 2009, of 26 *An. labranchiae *tested, three females contained oocysts (two carrying one oocyst and one carrying three) compared with 89% for control *An. gambiae *and, on Day 15, PCR detected sporozoites in the salivary glands of three other individuals (different from those carrying oocysts) [48]. One of the five identified *An. melanoon *individuals contained an oocyst.

In contradiction of the belief that European anophelines are refractory to tropical strains of *P. falciparum *[45,49], results of the current study show that *An. labranchiae *from Corsica is capable of replicating and even transmitting, a strain of *P. falciparum *from Africa (although infection levels are low). With respect to competence for *P. vivax*, it has been shown that members of the Maculipennis species complex are able to develop sporozoites of this species [50-53] after an infected blood meal, but in absence of reliable *P. vivax *gametocyte culture, experimental competence is difficult to assess.

### Malaria risk in Corsica

In Corsica, indigenous malaria disappeared (apart from in very minor foci) after the Second World War, as elsewhere in Europe. Although the last diagnosed cases were due to *Plasmodium vivax*, *P. falciparum *also used to be common [17], accounting for 48.9% of all cases of malaria documented in a 1947 epidemiological survey of schoolchildren (n = 1880) throughout Corsica. By way of comparison, before the Second World War (and the beginning of malaria eradication programmes), *P. falciparum *accounted for 26.3% of malaria cases in Romania [54]. Parasite prevalence in Corsica dropped sharply over the following years--from 23.4% in 1947 to 1.5% (1948), 1% (1949) and then 0.5% (1950)--as a result of malaria control programmes; by 1950, *P. falciparum *accounted for just 2% of all malaria cases [55].

The disappearance of malaria is due to synergistic improvements in a number of fields, notably improved diagnosis, more effective drug treatments (e.g. quinine), the elimination of larval larval habitats using environmental measures, vector control using insecticides (mainly DDT and later HCH, Chlorpyrifos, Fenitrothion, pyrethroids), and larvivorous fish, such as *Gambusia sp*., improvements in human houses and the distancing of animals from living quarters. The fact that only *P. vivax *persists during a phase of eradication is a classic pattern which is mainly due to this parasite's sporogonic cycle, being three to five days shorter than that of *P. falciparum *at equivalent temperature [56].

Entomological risk, and thus epidemiological risk if the parasites are present in human carriers, is often estimated through the basic reproduction rate (R0) of *P. vivax *or *P. falciparum *[56]. The basic reproductive rate is the total number of malaria cases derived from one infective case that the mosquito population would distribute to man. R0 must equal at least 1 for the disease to persist or spread. R0 is the product of the vectorial capacity (C), the infectiousness of vectors to humans (b) and humans to vectors (c), and the human infectious period (1/r): R0: (ma^2 ^p^n^/-ln p) . bc .1/r.

Vectorial capacity (C: ma^2 ^p^n^/-ln p) is defined as 'the average number of inoculations with a specified parasite, originating from one case of malaria in unit time that the population would distribute if all the vector females biting the case become infected'.

m is the vector-host ratio (i.e. the anopheline abundance in relation to humans)

a is the human feeding rate: the number of human bites per mosquito, per day.

p is the daily survival rate (i.e. the probability of a mosquito surviving one whole day).

n is the number of days required for *Plasmodium *sporogonic development (i.e. the time necessary for parasites to complete development from ingested gametocytes in the bloodmeal to sporozoites in the salivary glands).

c is closely linked to the vectorial competence of mosquitoes to *Plasmodium *species.

In R0, two parameters are more important than the others: 'a' and 'p'. 'a', the human feeding rate is squared because the mosquito needs to bite twice to transmit the parasite, firstly to become infected, and secondly to infect, after completion of sporogonic development. For this reason, a small change in mosquito feeding preference, or access to humans, will have a serious impact on malaria transmission. Even more importantly, 'p', the survival rate, owing to its power n, has a disproportionate impact on transmission. As the sporogonic development of *Plasmodium *is relatively long (e.g. for *P. falciparum*: 12- 30 days, *P. vivax*: 9- 30 days, depending on temperature) relative to mosquito lifespan (about 2-4 weeks for *An. labranchiae *in summer [57]), mosquitoes can be infectious for only a very limited period.

The risk of R0 being greater than 1 in Corsica (i.e. the risk of new isolated indigenous cases of malaria, of foci or even re-emergence, and establishment of *P. falciparum *and *P. vivax *malaria) therefore depends on a combination of several factors: parasites would have to be imported; competent anophelines for these parasites would have to be present; temperatures would have to be conducive to parasite growth inside mosquitoes within a time frame matched to the vector's life expectancy; anopheline biology--density, longevity, trophic behaviour--would have to be compatible with vector transmission; and finally, humans would have to be sufficiently exposed to *Anopheles *[58].

To summarize, a human carrying gametocytes would have to be bitten by a competent female anopheline, which would then have to survive for at least two more weeks and then feed on another human being. Estimating the risk of indigenous malaria corresponds to the probability of this series of events.

*Plasmodium*--almost exclusively *P. falciparum*--is regularly introduced into mainland France, mainly from Africa [59]. A survey published in 2005 [60] on malaria surveillance, based on cross-referencing various sources of information (Centre national de référence de l'épidémiologie du paludisme d'importation et autochtone, Paris; Centre national de référence de la chimiorésistance du paludisme, Paris; Programme médicalisé de système d'information [PMSI]) identified at least 46 cases of imported malaria in Corsica between 1999 and 2002, mostly due to *P. falciparum *(with just three cases due to *P. vivax*). Of these, 61% were identified between July and November, the season in which *Anopheles *are active and the temperature is conducive to rapid sporogonic development.

In Corsica, a number of anopheline species could possibly act as a vector, but *An. labranchiae *is the leading candidate by virtue of its historic role in the transmission of *P. falciparum*, *P. vivax *and *Plasmodium malariae*, its experimentally demonstrated vector competence, its biology, and its widespread distribution across the island. Its populations are monitored at regional level and controlled by local mosquito control agencies. In the framework of vector control, potential larval habitats for *Anopheles *(all species) are identified and sites that are confirmed as being active are treated on a weekly basis with *Bacillus thuringiensis israelensis. Anopheles labranchiae *can nevertheless persist at high densities in certain biotopes if they are not adequately treated. In these areas, the mosquito may be anthropophilic but, in practice, it hardly ever comes into contact with human beings.

The situation in Corsica is, therefore, different from that in the rice-growing regions of Tuscany where, along the western coast of Calabria, the vector competence of *An. labranchiae *was recently estimated at between 7.3 and 26 for *P. falciparum*, and between 8.3 and 32.5 for *P. vivax*, with peak biting rates of over 200 mosquitoes per person per night [20]. Other anopheline species would seem to be less likely candidates although most can support the replication of *P. falciparum *in the laboratory and have been known to act as vectors in other parts of Europe.

## Conclusion

In Corsica, there are *Anopheles *species that have been excellent vectors for *Plasmodium *within the last 60 years. These vectors persist in several formerly malarial tourist areas. These mosquitoes, especially *An. labranchiae*, are competent vectors in the laboratory. Human carriers of *P. falciparum *and *P. vivax *have been identified on the island every year, and rising levels of traffic and tourism will no doubt sustain the influx of parasites. The theoretical risk of indigenous malaria and malaria foci is not therefore zero. However, contact between humans and *Anopheles *is now very limited, carriers of *Plasmodium *are effectively detected and treated, tourists are better informed about prevention and prophylaxis, there are generalised systems for monitoring and controlling *Anopheles *populations across the whole island, and mosquito repellent products are in widespread use: taken together, all these factors make the risk negligible. Even in the areas at highest risk, i.e. the coastal regions where malaria was common before the eradication phase, areas of greatest human traffic and where *An. labranchiae *density remains high, the basic reproduction rate should remain well below 1, ensuring that indigenous malaria will remain restricted to rare, isolated cases.

## Competing interests

The authors declare that they have no competing interests.

## Authors' contributions

CT participated in designing of the study, in field work and carried out the molecular genetic studies, HB participated in field work and collecting historical data, GLG participated in field work, molecular studies and in collecting historical data, ITL conducted experimental work on vector competence, NR and DC participated in field work, DF conceived of the study, and participated in its design and coordination. All authors participated in writing the manuscript. All read and approved the final manuscript.
